# Anti-citrullinated protein antibodies are associated with osteopenia but not with pain at diagnosis of rheumatoid arthritis: data from the BARFOT cohort

**DOI:** 10.1186/s13075-019-1833-y

**Published:** 2019-02-04

**Authors:** Ingiäld Hafström, Sofia Ajeganova, Kristina Forslind, Björn Svensson

**Affiliations:** 1 Division of Gastroenterology and Rheumatology, Department of Medicine, Huddinge, Karolinska Institutet, and Rheumatology unit R92, Karolinska University Hospital, Stockholm, SE 141 86 Sweden; 2 Division of Gastroenterology and Rheumatology, Department of Medicine, Karolinska Institutet, Huddinge, Sweden; 3 Rheumatology Division, Universitair Ziekenhuis Brussel, Vrije Universiteit Brussel, Brussels, Belgium; 4 Department of Clinical Science, Section of Rheumatology, Faculty of Medicine, Lund University, Lund, Sweden; 5 Department of Research Education, Skånevård Sund, Region Skåne, Helsingborg’s hospital, Helsingborg, Sweden; 6 Department of Clinical Science, Section of Rheumatology, Faculty of Medicine Lund University, Lund, Sweden

**Keywords:** Rheumatoid arthritis, Anti-citrullinated protein antibodies, Bone mineral density, Pain

## Abstract

**Background:**

Anti-citrullinated protein antibodies (ACPA) have been suggested to have a potential role in both bone loss and pain in rheumatoid arthritis (RA), based on studies in vitro and in animal models. Here we addressed if anti-cyclic citrullinated (anti-CCP) antibodies were associated with osteopenia or pain in patients with RA, at the time for diagnosis.

**Methods:**

Baseline data from the BARFOT (Better Anti-Rheumatic PharmacOTherapy) cohort, which consists of patients with RA with a disease duration of 1 year or less, were analyzed. To be included, they should have been assessed by anti-CCP, dual-energy X-ray absorptiometry (DEXA) of lumbar spine and hip, and/or digital X-ray radiogrammetry (DXR) of the metacarpal bones. Osteopenia was defined as a z-score < − 1 SD. Pain VAS > 40 mm, was defined as patient unacceptable pain. Multiple logistic regression analyses were performed to assess whether anti-CCP was independently associated with osteopenia or unacceptable pain.

**Results:**

Of the 657 patients, 65% were women, 58% were anti-CCP positive, 37% had osteopenia in the lumbar spine, and 29% had osteopenia in the hip. Sixty-one percent had unacceptable pain at diagnosis. Patients positive for anti-CCP had significantly more frequently osteopenia in the femoral neck and Ward’s triangle compared with anti-CCP-negative patients (*p* = 0.016 and 0.003, respectively). This difference was found in men at any anti-CCP titer, but in women, osteopenia in these hip locations was found only in those with high anti-CCP titers (> 500 IU/ml). Anti-CCP was not associated with osteopenia in the lumbar spine or the metacarpal bones. In multiple logistic regression analyses, anti-CCP was independently associated with osteopenia in the femoral neck and/or Ward’s triangle but not with unacceptable pain. Instead, inflammatory variables were independently associated with unacceptable pain.

**Conclusion:**

These data show that in patients with early RA, anti-CCP positivity was independently associated with osteopenia in the femoral neck and/or Ward, but not in the lumbar spine. In our patients, we could not confirm a recently suggested association between anti-CCP antibodies and pain. Further studies are necessary to explore the possible clinical relevance of interactions between ACPA, bone, and pain found in vitro and in animal models.

## Introduction

Many patients with rheumatoid arthritis (RA) have osteopenia and pain as prominent features, often not associated with clinical joint inflammation [1–4]. Possible mechanisms responsible for inflammation-independent osteopenia or pain have been proposed, among these a potential role for anti-citrullinated protein antibodies (ACPA) [5].

Presence of ACPA analyzed by the anti-cyclic citrullinated antibodies test (anti-CCP2) has been found in 0.8% of the general non-RA population. In these individuals, joint pain was significantly more frequent than in the anti-CCP-negative group [6]. Furthermore, anti-CCP-positive individuals with unspecific musculoskeletal complaints, including joint pain, had a high risk of developing RA [7]. It should though be noted that anti-CCP-negative RA exists, a disease with a heterogenous course, in which the prognosis also could be severe.

ACPAs detectable as anti-CCP antibodies, as well as other autoantibodies, may appear in blood prior to the clinical onset of RA [8, 9]. The ACPA immune response starts in a restricted manner and the number of recognized citrullinated peptides increases in the period preceding disease onset [10–12]. With time, also the ACPA levels increase, markedly 2–4 years prior to a diagnosis of RA, alongside a rise in inflammatory cytokine levels [13, 14].

Monoclonal ACPA derived from patients with RA can in vitro cause differentiation of human osteoclasts and induce trabecular bone loss in mice [15, 16]. Osteoclasts stimulated by ACPA secrete, as the main mediator, the cytokine interleukin (IL)-8. This cytokine is not only an autocrine growth factor for osteoclasts but can also induce pain in mice in the absence of inflammation, probably by sensitizing or activation of the sensory neurons [16, 17]. These studies provided a possible role of ACPA in both bone loss and pain.

In healthy anti-CCP-positive individuals, cortical bone loss in the metacarpophalangeal joints (MCP) has been reported [18]. Furthermore, in ACPA-positive subjects with arthralgia, trabecular thinning of MCP has been observed [19]. However, in the early treatment-naïve RA, bone mineral density (BMD) is preserved in most patients [20]. A recent study has shown that anti-CCP positivity is associated with reduced BMD, especially of the trabecular bone in the lumbar spine [21]. Interestingly, anti-CCP at any level in that study negatively affected the spine BMD while BMD in the hip was affected by high anti-CCP titers only [21].

The potential association of ACPA and pain intensity has however not been studied.

The aim of the present study was to assess whether ACPA measured as anti-CCP was associated with BMD or pain in the initial stage of RA and thereby to have a possible role in bone loss and pain perception. For this study, we took advantage of the cohort of early RA patients recruited to the Better Anti-Rheumatic PharmacOTherapy (BARFOT) cohort in 1993–1999, provided that the patients had measurements of BMD in the hip and lumbar spine and/or metacarpophalangeal bone.

## Patients and methods

### Study design and population

The BARFOT cohort is recruited from six centers, which cover both urban and rural referral areas. It is an observational study designed to investigate the course and long-term prognosis of RA. The inclusion criteria were a diagnosis of RA according to the ACR classification criteria [22] and a disease duration of 12 months or less [23]. In all 909 patients, 18 to 80 years of age, not previously treated with disease-modifying anti-rheumatic drugs (DMARDs) or glucocorticoids, were recruited from October 1993 to November 1999.

For this study, patients were eligible if they had measured anti-CCP, bone mineral density (BMD) with dual-energy X-ray absorptiometry (DEXA) (*n* = 557) and/or digital X-ray radiogrammetry (DXR) of the metacarpal bones on hand radiographs (*n* = 391) at baseline. A total of 657 patients satisfied these criteria. The patients with missing data, 252 patients, had similar baseline characteristics as those included into this study (data not shown).

### Disease activity and physical function

Disease activity was assessed by C-reactive protein (CRP) and the Disease Activity Score calculated on 28 joints (DAS28, range 0–9.4) [24]. For patients assessed early in the BARFOT study, their original DAS values at inclusion were transformed to DAS28 using the formula described by van Gestel et al. [25].

Pain was measured on a visual analogue scale (VAS) (0–100 mm, least to worst). Patient unacceptable pain state was defined as pain VAS > 40 mm [26].

The Swedish version of the Stanford Health Assessment Questionnaire (HAQ) was used to measure daily life function [27].

### Laboratory analyses

Plasma and serum samples were stored at − 70 °C until assay. Antibodies to cyclic citrullinated peptides (anti-CCP) were detected using the ELISA CCP2 test (Euro-Diagnostica, Malmö, Sweden) at three medical centers, and positive anti-CCP was defined as > 25 IU/ml. Rheumatoid factor was analyzed by agglutination test at different centers, while only serostatus was used. A titer of 20 IU/ml was regarded as positive.

### Bone mineral density

BMD at the lumbar spine (L1 and L2–L4) and at the left hip (femoral neck, Ward’s triangle, and greater trochanter) was measured by dual-energy X-ray absorptiometry (DEXA) with a Lunar densitometer. DEXA-BMD was expressed as the number of standard deviations (SD) from the mean of healthy age- and sex-matched people, the z-score. The values were obtained from Lunars combined European/US reference population (Expert-XL software version 1.7, Lunar Corp. Madison, USA, 1998). The z-scores were used to minimize the effect of age and gender. The lowest z-score in the lumbar spine is given, and for the hip area, separate z-scores for femoral neck, Ward’s triangle, and trochanter were given. Reduced bone mass was defined as a z-score ≤ 1.0 SD below the mean value measured by the DEXA.

Digital X-ray radiogrammetry (DXR) was used to measure cortical BMD of the three middle metacarpal bones on hand radiographs at baseline. Analogue X-ray films of hands were digitized using a Vidar Diagnostic Pro plus, 300 dpi, 12 bit (VIDAR Systems Corp., Herndon, VA, USA). DXR-BMD was measured on the digitized images by DXR-online (Sectra, Linköping, Sweden). The technique has been described in detail previously [28, 29]. In short, this method provides a BMD estimate in grams per square centimeter based on an automated analysis of the geometry and texture of the cortical bone of the three middle metacarpals. The estimated DXR-BMD was compared to a reference database of healthy age- and sex-matched individuals to calculate z-scores. Osteopenia was defined as z-score ≤ 1.0 SD below the mean value measured by DXR [30].

### Statistics

Statistical analyses were performed using IBM SPSS statistical software v21.0. To test differences between groups, the *T* test was used for independent groups and the chi-square test for proportions. Correlations were performed by Spearman’s test.

Multiple logistic regression analyses were performed to assess whether anti-CCP independently predicted osteopenia or unacceptable pain. The models initially included possible confounders and other potential independent predictors, i.e., age, gender, disease duration, smoking habits, body mass index (BMI), DAS28, CRP, and HAQ, but not the rheumatoid factor (RF) due to high correlation with anti-CCP (*r* = 0.7). Variables failing to meet a threshold of *p* < 0.25 were removed.

## Results

### Patient characteristics and anti-CCP status

A total of 657 patients with early RA, for whom baseline anti-CCP and BMD measurements were available, were included. Mean (SD) age was 57 (15) years, 58% were anti-CCP positive and 60% RF positive. Of the study population, 65% were women, of whom 58% were postmenopausal. All patients had active disease, DAS28 mean (SD) 5.2(1.2), and mean (SD) pain VAS 47 (24) mm, and 61% had pain VAS > 40 mm. None of the patients were treated with bisphosphonates or vitamin D supplements.

Table 1 shows the characteristics of the patients according to anti-CCP status. Anti-CCP-positive patients were more often ever smokers, were positive for RF, and had higher ESR, CRP, and pain (only men) but less tender joints (only women) than anti-CCP-negative patients.

**Table 1:** Patients and baseline disease characteristics split by anti-CCP status and gender

	All patients			Women			Men		
	Anti-CCP		*p* value	Anti-CCP		*p* value	Anti-CCP		*p* value
	Positive	Negative		Positive	Negative		Positive	Negative	
	*n* = 378	*n* = 279		*n* = 244	*n* = 184		*n* = 134	*n* = 95	
Age, years	56	58	0.23	55	54	0.33	58	65	**0.01**
Disease duration, months	6	6	0.06	6	6	0.13	6	6	0.23
Ever smokers, %	66	55	*0.006*	61	51	*0.046*	74	63	0.07
BMI, kg/m^2^	25.4	25.5	0.60	24.9	25.5	0.23	26.0	25.5	0.36
RF-positivity, %	88	21	*0.001*	85	16	*0.001*	86	14	*0.001*
DAS28	5.3	5.2	0.19	5,3	5,3	0.66	5.2	4.9	0.10
Swollen joints, *n*	10	11	0.053	10	11	0.08	11	11	0.39
Tender joints, *n*	7	9	*0.007*	8	10	*0.004*	7	7	0.66
Patient global assessment VAS (0–100), mm	46	45	0.71	49	48	0.69	41	40	0.83
ESR, mm	41	31	*0.001*	41	30	*0.001*	41	32	*0.001*
CRP, mg/l	24	16	*0.001*	20	14	*0.001*	29	24	*0.040*
Pain VAS (0–100), mm	49	45	*0.020*	51	47	0.15	47	40	*0.042*
Pain VAS > 40 mm %	65	56	*0.021*	66	59	0.13	62	50	0.07
HAQ (0–3)	1.0	1.0	0.17	1.2	1.0	0.10	0.9	0.8	0.82

*p* values denote differences between groups. The values are mean (normally distributed) unless otherwise stated. Italicized *p* values are significant. Anti-CCP positivity titer > 25 IU/ml

*BMI* body mass index, *RF r*heumatoid factor, *DAS28* 28 joints-Disease Activity Score, *ESR* erythrocyte sedimentation rate, *CRP* C-reactive protein, *VAS* visual analogue scale, *HAQ* Health Assessment Questionnaire

The same differences in clinical characteristics were present when comparing patients with anti-CCP titer above 500 IU/ml with anti-CCP-negative patients, except for pain and tender joints which were similar (data not shown).

### Anti-CCP and bone mineral density

A total of 557 patients had DEXA performed. Reduced bone mass, defined as osteopenia by DEXA, was found in 37% of the patents in the lumbar spine (35% in women and 41% in men) and in 29% in the femoral neck, Ward, or trochanter (28% in women and 30% in men).

Patients positive for anti-CCP had significantly more often osteopenia in the femoral neck and Ward than anti-CCP-negative patients, but when separating the patients per gender, this difference was found only in men (Table 2).

**Table 2:** Baseline bone mineral density, measured by DEXA and DXR, by anti-CCP status and gender

	All patients			Women			Men		
	Anti-CCP		*p* value	Anti-CCP		*p* value	Anti-CCP		*p* value
	Positive	Negative		Positive	Negative		Positive	Negative	
DEXA									
Lumbar spine									
z-score, mean (SD)	− 0.63 (1.25)	− 0.46 (1.39)	0.14	− 0.60 (1.17)	− 0.53 (1.25)	0.65	− 0.70 (1.38)	− 0.31 (1.62)	0.09
Osteopenia, %	38	36	0.54	35	35	0.99	44	36	0.34
Femoral neck									
z-score, mean (SD)	0.05 (1.24)	0.03 (0.96)	0.85	0.17 (1.29)	− 0.11 (0.88)	0.07	− 0.14 (1.13)	0.31 (1.06)	*0.025*
Osteopenia, %	18	9	*0.016*	14	9	0.30	25	8	*0.017*
Femoral Ward									
z-score, mean (SD)	− 0.07 (1.14)	0.20 (1.16)	*0.028*	0.00 (1.14)	0.11 (1.10)	0.46	− 0.19 (1.14)	0.38 (1.25)	*0.009*
Osteopenia, %	21	9	*0.003*	19	10	0.70	23	6	*0.011*
Femoral trochanter									
z-score, mean (SD)	0.12 (1.18)	0.18 (1.04)	0.64	0.25 (1.18)	0.05 (0.97)	0.19	− 0.10 (1.17)	0.43 (1.14)	*0.016*
Osteopenia, %	18	14	0.29	15	17	0.66	23	6	*0.018*
DXR, metacarpal									
z-score, mean (SD)	− 0.10 (1.09)	− 0.03 (1.13)	0.52	− 0.24 (1.15)	− 0.23 (1.12)	0.74	0.11 (0.96)	0.44 (1.09)	*0.047*
Osteopenia, %	22	20	0.65	26	26	1.00	15	8	0.30

*p* values denote differences between groups, and italicized values are significant. Anti-CCP positivity titer > 25 IU/ml

*DEXA* dual-energy X-ray absorptiometry, assessed in 557 patients, *DXR* digital X-ray radiogrammetry, assessed in 391 patients

We therefore compared the women who were 50 years or older with those below 50 (median menopausal age was 49.1 years). In these two age subgroups, z-scores in the hip compartments did not differ between anti-CCP-positive and anti-CCP-negative patients (data not shown), except for anti-CCP-positive women 50 years or older who had more often osteopenia in Ward than those negative, 24% and 7% respectively (*p* = 0.009).

Lumbar BMD was not affected by anti-CCP status, neither in women nor in men (Table 2), not even when controlling for disease activity measured by DAS28 (data not shown).

To explore further the influence of anti-CCP on osteopenia, we performed analyses of the effects of high anti-CCP titers on bone mass. For these analyses, we applied a titer above 500 IU/ml (median titer was 475 IU/ml).

A total of 180 patients had an anti-CCP titer above 500 IU/ml, 103 women and 77 men. Both women and men with such a high titer had significantly higher frequency of osteopenia in the femoral neck and/or Ward than those who were anti-CCP negative (Table 3). Of interest, this high anti-CCP titer was not associated with osteopenia in the lumbar spine in any gender.

**Table 3:** Baseline bone mineral density measured by DEXA and DXR, by groups with anti-CCP > 500 IU/ml and anti-CCP negative

	All patients			Women			Men		
	Anti-CCP		*p* value	Anti-CCP		*p* value	Anti-CCP		*p* value
	> 500 IU/ml	Negative		> 500 IU/ml	Negative		> 500 IU/ml	Negative	
	*n* = 180	*n* = 277		*n* = 103	*n* = 183		*n* = 77	*n* = 94	
DEXA									
Lumbar spine									
z-score, mean (SD)	− 0.57 (1.22)	− 0.48 (1.36)	0.53	− 0.53 (1.19)	− 0.53 (1.25)	0.97	− 0.63 (1.27)	− 0.37 (1.55)	0.32
Osteopenia, %	33	36	0.56	31	36	0.52	35	37	0.85
Femoral neck									
z-score, mean (SD)	− 0.10 (1.10)	0.02 (0.96)	0.32	− 0.04 (1.18)	− 0.10 (0.89)	0.69	− 0.19 (0.99)	0.28 (1.05)	*0.027*
Osteopenia, %	23	9	*0.002*	22	9	*0.028*	24	8	*0.036*
Femoral Ward									
z-score, mean (SD)	− 0.19 (1.12)	0.18 (1.16)	*0.01*	− 0.19 (1.23)	0.10 (1.10)	0.13	− 0.20 (0.96)	0.35 (1.25)	*0.020*
Osteopenia, %	22	9	*0.004*	23	10	*0.035*	20	6	*0.043*
Femoral trochanter									
z-score, mean (SD)	− 0.06 (1.16)	0.16 (1.02)	0.13	− 0.03 (1.30)	0.05 (0.98)	0.68	− 0.10 (0.93)	0.37 (1.08)	*0.032*
Osteopenia, %	20	14	0.18	23	17	0.40	17	7	0.13
DXR, metacarpal									
z-score, mean (SD)	0.01 (1.08)	− 0.03 (1.13)	0.80	− 1.13 (1.23)	− 0.29 (1.12)	0.40	0.13 (0.91)	0.45 (1.01)	0.09
Osteopenia, %	17	20	0.34	23	26	0.40	12	8	0.40

*p* values denote differences between groups, and italicized values are significant

*DEXA* dual-energy X-ray absorptiometry, *DXR* digital X-ray radiogrammetry

We further analyzed if anti-CCP status was associated with cortical bone mass measured by DXR in the metacarpophalangeal bones. The frequency of osteopenia was not influenced by positive anti-CCP or by anti-CCP titers above 500 IU/ml, neither in women nor in men (Tables 2 and 3).

### Predictors of osteopenia or pain

Multiple logistic regression analyses with osteopenia in the femoral neck and/or Ward as the dependent variable showed that anti-CCP positivity was independently associated with osteopenia (Table 4).

**Table 4:** Multiple logistic regression with osteopenia of the femoral neck and/or Ward as the dependent variable

	B	S.E.	Sig.	Exp(B)	95% CI for EXP(B)	
					Lower	Upper
Anti-CCP positivity	0.719	0.277	*0.009*	2.053	1.193	3.535
Female gender	−0.182	0.262	0.487	0.834	0.499	1.393
Constant	−1.611	0.285	0.000	0.200		

Anti-CCP positivity = > 25 IU/ml. Italicized value is significant

If high levels of anti-CCP (above 500 IU/ml) were included in the model instead of the categorical anti-CCP variable, the results became similar (data not shown).

When RF serostatus (yes/no) was included in the model instead of anti-CCP serostatus, no association with osteopenia in femoral neck/ward was found (data not shown). If both categorical anti-CCP and RF were included together, they were associated with osteopenia but with a lower odds ratio than anti-CCP alone (1.79 versus 2.05).

Multiple regression analyses to explain unacceptable pain showed that inflammation (DAS28) and HAQ, but not anti-CCP positivity, were independently associated with unacceptable pain (Table 5). Similar results were obtained if high-level anti-CCP (above 500 IU/ml) was included in the model instead of the categorical anti-CCP variable (data not shown).

**Table 5:** Multiple logistic regression with unacceptable pain as the dependent variable

	B	SE	Sig.	Exp(B)	95% CI for EXP(B)	
					Lower	Upper
Age	− 0.009	0.006	0.159	0.991	0.979	1.003
Disease duration, months	− 0.042	0.030	0.159	0.959	0.905	1.017
Anti-CCP positivity	0.293	0.184	0.113	1.340	0.934	1.923
DAS28	0.267	0.088	*0.002*	1.306	1.100	1.552
HAQ	1.225	0.180	*0.000*	3.404	2.393	4.844
Constant	− 1.518	0.578	0.009	0.219		

Anti-CCP positivity = > 25 IU/ml. Italicized values are significant. Unacceptable pain defined as VAS pain > 40 mm

*DAS28* 28 joints-Disease Activity Score, *HAQ* Health Assessment Questionnaire

## Discussion

In the present study of patients with early RA, osteopenia in lumbar spine and/or hip was found in about one third of the patients. Patients positive for anti-CCP had a higher frequency of reduced bone mass, osteopenia, in the femoral neck/Ward’s triangle measured by DEXA than those who were anti-CCP negative. Further, anti-CCP positivity was independently associated with osteopenia in these hip locations. This observation corresponds to a recent report that has suggested a possible direct role of ACPA on bone metabolism. However, anti-CCP was not associated with high pain perception in this study.

The frequency of osteopenia was of the same magnitude as previously reported in early RA [20] and was, at diagnosis, not associated with disease-related variables, such as disease activity or functional impairment, variables known to be associated with systemic bone loss in established RA [1]. However, RA-related autoimmunity may induce bone loss in absence of overt inflammation. Thus, altered bone metabolism has been shown already in the preclinical phase of anti-CCP-positive individuals [31].

In the present study, the presence of anti-CCP was not associated with osteopenia in the lumbar spine, which might depend on the fact that BMD measurement of the lumbar spine by DEXA is influenced by its sensitivity to degenerative and osteoarthritic changes, which can result in incorrect values. Another possibility is that anti-CCP autoantibodies early in the disease do not yet contribute to systemic osteopenia in bone remote from the inflamed joints. Contrary to our results, in a cohort with early RA from Italy, an association between anti-CCP positivity and lumbar spine osteopenia has been reported [21]. However, there are significant differences between the cohorts. Thus, in Bugatti’s cohort, fewer patients were anti-CCP and RF positive, 43 and 43%, respectively, compared with 58 and 60% in the present patients, and only 26% had osteopenia in the lumbar spine compared with the present 37%. Furthermore, only 69% in Bugatti’s cohort satisfied the ACR 1987 criteria for RA. These cohort differences make comparisons of the results problematic.

Also, in a Spanish cohort of early arthritis (a PEARL study) with 38% anti-CCP-positive patients, an association between anti-CCP positivity and low bone density in lumbar spine was demonstrated [32]. This cohort included both patients fulfilling the ACR/EULAR 2010 RA criteria (55%) and patients with undifferentiated arthritis, spondyloarthropathies, osteoarthritis, connective tissue disorders, and miscellaneous conditions. Besides the heterogenous population, the fact that a good 20% of the patients were on glucocorticoid therapy makes the PEARL and BARFOT cohorts difficult to compare. In a randomized study of early addition of prednisolone 7.5 mg/day to the first DMARD in patients diagnosed with RA, we have shown that the effect of glucocorticoids on the bone can be both beneficial (hip) and deleterious (lumbar spine) [33].

The differences in associations between anti-CCP and BMD in the different hip compartments may depend on the fact that their bone mineral density declines differently with age. Thus, in elderly individuals, BMD in Ward’s triangle is lower than that in the other hip compartments [34] and may be more influenced by anti-CCP-related autoimmunity. This is in line with the finding of bone loss in the cortical bone of the total hip only by high anti-CCP titers in the Bugatti cohort [21]. However, anti-CCP positivity was associated with osteopenia in both total hip and femoral neck in the PEARL study [32].

In men, anti-CCP at any titer was associated with osteopenia in the hip, while in women this association was found only at higher titers of anti-CCP. It is well-known that ACPAs develop several years ahead of the diagnosis of RA and that the number of citrullinated peptides increases over time in a random manner as well as the antibody titer [11]. However, none of the studies describing this phenomenon has separated the results per gender [8–13]. Furthermore, gender was not considered in analyses of the titer-dependent role of ACPA on bone loss in the hip in established RA [35, 36].

In the present study, women and men had the same frequency of anti-CCP positivity as well as the same frequency of anti-CCP titer above 500 IU/ml. However, we have no information of whether epitope spreading and the number of ACPA isotypes differed. Besides, the identity of the molecule(s) on osteoclasts targeted by ACPA remains unresolved [5]. Therefore, it is so far not known if different antigens are targeted in a different manner in women and men with RA. The finding of more likely osteopenia in Ward in women older than 50 years compared with women younger than 50 years in our study suggests the importance of studying postmenopausal systemic bone remodeling in addition to autoantibody-dependent bone changes.

The association between anti-CCP positivity and osteopenia in the femoral neck/Ward in the present study does not allow any inferences of causality but fits well with recent reports linking ACPA to autoantibody-mediated bone loss [15, 16]. However, also other autoantibodies may contribute to bone loss in patients with RA. Thus, the association between anti-CCP positivity and BMD loss was reported to be enhanced in the presence of concomitant high levels of RF autoantibodies [21]. In our study, RF per se was not associated with osteopenia; however, because of variation in the used assays, dose-dependent RF effect was not examined.

Interestingly, anti-carbamylated protein (anti-CarP) autoantibodies, additive to ACPA and RF, have been associated with more severe radiographic progression in patients with early RA [37]. Thus, several autoantibodies might have pathogenic effect on osteoclasts. Recently, it has been reported that in the anti-CCP-positive patients with early arthritis, high titers of anti-CarP have contributed to finding of osteopenia in both lumbar spine and hip [38].

Neither presence of anti-CCP nor high titers of anti-CCP were associated with metacarpal (MCP) cortical bone osteopenia assessed by DXR. This might be explained by the lower turnover rate in the cortical bone compared to the trabecular bone. A study performed with micro computed tomography has shown thinning and fenestration of the cortical bone in the MCP in anti-CCP-positive healthy individuals [18]. In the studies of RA, loss of the metacarpal cortical bone, assessed by DXR, occurs early upon the diagnosis of both undifferentiated arthritis and RA [29, 39]. In these studies of early RA, the effect of ACPA on bone loss was not evaluated. On the other hand, in established disease with a disease duration of 2 to 4 years, elevated anti-CCP levels independently predicted loss of the cortical hand bone during a study period of 5 years [40].

Anti-CCP positivity was not associated with unacceptable pain in this study. Instead, inflammation seemed to play an important role in pain experience before treatment initiation. In RA, pain is mostly attributed to inflammation, but non-inflammatory pain exists and may confound disease activity assessments by, e.g., DAS28 [41].

In the present study, the patient reported pain was assessed by VAS, which does not specify the kind or character of pain. The pain is suggested to be of peripheral nociceptive origin, possibly with peripheral sensitization as peripheral sensitization due to inflammation can amplify pain [42]. The lack of an association of anti-CCP with unacceptable pain may depend on different effects of ACPA on inflammatory-dependent and non-inflammatory pain.

Induction of pain by ACPA has been reported in mice [17], and it has further been suggested that the arthralgia preceding RA in ACPA-positive individuals may be a direct consequence of osteoclast activation linked to certain ACPAs via an IL-8-dependant pathway [5].

The role of ACPA in the pathogeneses of RA remains unclear. In many patients, treatment with disease-modifying agents reduces inflammation and pain, whereas the level of anti-CCP remains stable over time [43]. However, the ACPA response differs markedly across specificities, which suggests that only some ACPAs are pathogenic [43].

There are limitations in this study. First, this is an observational study not designed to study causal relationships. Furthermore, the possibility of subgroup analyses was limited by the relatively small number of patients available. It should also be noted that DEXA measures only bone mass and not bone microstructure nor biomechanical properties of bone. Although information on hormone replacement therapy was lacking, it should not influence our findings on the association between anti-CCP positivity and osteopenia as the postmenopausal women were similarly represented among anti-CCP-positive and anti-CCP-negative RA patients.

To recall, the present analysis of the second-generation anti-cyclic citrullinated peptide (anti-CCP2) does not detect all anti-citrullinated protein/peptide antibodies (ACPAs) [44]. Thus, a subgroup of anti-CCP-negative patients in our study may still display ACPA reactivity. Furthermore, the lack of statistically proven association of anti-CCP positivity with unacceptable pain should be interpreted with caution as a negative finding may depend not only on sample size, definition of pain, and different effects of ACPA on inflammatory-dependent and non-inflammatory pain, but also on co-existence of other autoantibody systems, not measured here, possibly affecting pain. However, considering pain burden important for the patients, the outcome of unacceptable pain in this study appears to be relevant.

## Conclusion

Our data show that in patients with early RA, anti-CCP positivity, independently of inflammation, is associated with osteopenia in the femoral neck and Ward, but not with unacceptable pain. Further studies are necessary to explore the possible clinical relevance of interactions between ACPA, bone remodeling, and pain reported in vitro and in animal models.

## Abbreviations

ACPA: Anti-citrullinated protein antibodies
Anti-CarP: Anti-carbamylated protein
Anti-CCP: Anti-cyclic citrullinated antibodies
BARFOT: Better Anti-Rheumatic PharmacOTherapy
BMD: Bone mineral density
BMI: Body mass index
CRP: C-reactive protein
DAS28: Disease Activity Score calculated on 28 joints
DEXA: Dual-energy X-ray absorptiometry
DMARD: Disease-modifying anti-rheumatic drug
DXR: Digital X-ray radiogrammetry
ESR: Erythrocyte sedimentation rate
HAQ: Health Assessment Questionnaire
IL: Interleukin
L: Lumbar spine
MCP: Metacarpophalangeal joint
PEARL: Princesa Early Arthritis Register Longitudinal
RA: Rheumatoid arthritis
RF: Rheumatoid factor
VAS: Visual analogue scale
